# Analysis of the Mating-Type Distribution and Fertility Variation in *Magnaporthe oryzae* Populations in China

**DOI:** 10.3390/jof12010040

**Published:** 2026-01-03

**Authors:** Han Yan, Jintao Liu, Han Xu, Jun Yang, Hai Dong

**Affiliations:** 1 Institute of Plant Protection, Liaoning Academy of Agricultural Sciences, Shenyang 110161, China; lnzbyh@163.com (H.Y.);; 2 Key Laboratory of Crop Pest Control in Liaoning Province, Shenyang 110161, China; 3 State Key Laboratory of Agricultural and Forestry Biosecurity, MARA Key Lab of Surveillance and Management for Plant Quarantine Pests, College of Plant Protection, China Agricultural University, Beijing 100193, China

**Keywords:** fertility, mating-type, *Magnaporthe oryzae*, rice blast, sexual reproduction

## Abstract

*Magnaporthe oryzae* exhibits significant genetic polymorphism in paddy fields. This study collected and isolated 832 single-spore isolates from major rice-producing areas of 17 provinces in six geographical regions across China, analyzing their mating-type distribution, fertility variation, and underlying mechanisms. Polymerase chain reaction (PCR) assays revealed a significantly higher proportion of the *MAT1-2* mating type (79.21%) than *MAT1-1* (20.79%), with severely skewed ratios in some regions. Correlation analysis indicated that mating-type distribution was significantly associated with effective accumulated temperature (≥10 °C). *MAT1-1* was predominantly concentrated in regions with 4500–7000 degree-days, whereas *MAT1-2* was mainly found in regions with 2500–5000 degree-days. Cross-culture fertility tests yielded an average fertility rate of 36.54% and mean perithecia production of 25.7 per isolate, suggesting generally low fertility, with *MAT1-2* isolates showing significantly higher fertility than *MAT1-1*. This study demonstrates that regional *M. oryzae* populations in China exhibit both mating-type imbalances and fertility deficiency, suggesting rare genetic recombination in natural populations and evolution primarily driven by asexual reproduction.

## 1. Introduction

Rice blast, which is caused by *Magnaporthe oryzae*, is the most devastating fungal disease of rice in the world [1,2,3]. *M. oryzae* is a heterothallic ascomycete with a bipolar mating system [4,5] that can infect a variety of gramineous crops and weeds [6]. As sexual reproduction is a key driver of genetic variation in fungi [7], mating type and fertility directly regulate the formation of the sexual stage and the frequency of genetic variation in field populations. Therefore, elucidating the distribution characteristics of mating-type genes and fertility in *M. oryzae* is crucial for understanding the dynamics of sexual reproduction in its populations. In fungal genetics research, artificial sexual hybridization is a classical approach for analyzing genetic traits [8]. Since the initial reports of the sexual stages in *M. grisea* and *M. oryzae* by Hebert and Kato, respectively [7,9], research has progressively advanced, with Kang et al. successfully cloning the genes controlling mating type in *M. oryzae* [10]. Subsequent studies have optimized experimental systems for the sexual stage [11,12,13], establishing a novel framework for analyzing the geographical differentiation and phylogeny of the pathogen based on its mating-type distribution and fertility characteristics. Similarly to other ascomycetes, mating type in *M. oryzae* is governed by the allelic genes *MAT1-1* and *MAT1-2* [14]. Sexual reproduction can only occur when both mating types are present within a population, although mating competence is influenced by numerous factors [15,16].

Approximately 30 million hectares of rice are cultivated across six geographical regions of China, where blast disease is a major epidemic [17]. China’s main rice-producing areas are distributed across diverse environments, including plains, mountainous areas, hills, and coastal regions. This geographical heterogeneity may drive genetic isolation in *M. oryzae*; however, the characteristics of geographical differentiation in its population genetic structure remain unclear. On the other hand, rice blast fungus has a very rich microbial community. Whether it originates from sexual reproduction has not been found in natural environments. To address these research gaps, our study systematically analyzed 832 *M. oryzae* isolates collected from 17 provinces across six geographical regions of China, aiming to reveal the population genetic characteristics of *M. oryzae* based on the dimensions of mating-type distribution patterns and variations in fertility.

## 2. Materials and Methods

### 2.1. Establishment of Disease Nursery and Sample Collection

A total of 832 single-spore isolates of *M. oryzae* were isolated from an universally blast-susceptible rice variety ‘LTH’ by using an approach described previously [18], in major rice-growing areas across 17 provinces, including 36 isolates from Heilongjiang (HLJ), 20 isolates from Jilin (JL), 336 isolates from Liaoning (LN), 46 isolates from Neimenggu (NMG), 14 isolates from Shandong (SD), 36 isolates from Jiangsu (JS), 32 isolates from Anhui (AH), 28 isolates from Zhejiang (ZJ), 37 isolates from Jiangxi (JX), 15 isolates from Fujian (FJ), 16 isolates from Henan (HeN), 31 isolates from Hubei (HB), 41 isolates from Hunan (HuN), 29 isolates from Sichuan (SC), 35 isolates from Yunnan (YN), 49 isolates from Guangdong (GD), and 31 isolates from Guangxi (GX) (Table A1). These areas span six geographical regions of China: northeast China (NEC), north China (NC), southwest China (SWC), east China (EC), central China (CC), and south China (SC) (Table A1). Two reference standard strains, P9 (*MAT1-1*) and P131 (*MAT1-2*), which are fertile *M. oryzae* strains with known mating types, were provided by the research group of Professor You-Liang Peng at China Agricultural University.

### 2.2. Identification of M. oryzae Mating-Type

Genomic DNA was extracted from *M. oryzae* using the CTAB method [19]. The mating type-specific primers (Table A3) were designed based on unique sequences of two mating-type genes, *MAT1-1* and *MAT1-2*, combined with the results in previous studies [20]. Mating types were identified by polymerase chain reaction (PCR) amplification with mating type-specific primers, followed by electrophoresis and the visualization of the gel imaging results. The PCR system (20 μL) consisted of 1 μL template DNA (quality was assessed with Nanodrop), 0.25 μL of each primer, 10 μL dNTP Mix (2.5 mM for each, Mei5bio, Beijing, China), 0.1 μL Taq polymerase (Biomarker, Beijing, China), and 7.9 μL ddH_2_O. The PCR amplification program was as follows: pre-denaturation at 95 °C for 3 min; 30 cycles of denaturation at 95 °C for 25 s, annealing at 50 °C for 25 s, and extension at 72 °C for 15 s; followed by a final extension at 72 °C for 10 min. A negative control (without template DNA) and positive controls (using the standard strains P9 and P131) were included in each run. During electrophoresis detection, 6 μL of PCR products were analyzed using 1.5% agarose gel in 1× TBE buffer. After staining with M5 nucleic acid dye (Mei5bio, Beijing, China), the results were visualized and recorded on a gel imaging system(CHEMIDoc, Hercules, CA, America).

### 2.3. M. oryzae Fertility Assessment

Fertility was evaluated using the cross-culture method [21]. Tested isolates and standard strains were simultaneously inoculated on 6 cm oatmeal tomato medium (OTA, 150 mL tomato juice, 20 g oatmeal and 15 g agar per liter) plates, with the isolates spaced 2 to 3 cm apart. Each plate contained one standard strain and two tested isolates of opposite mating types. Plates with paired standard strains served as positive controls, and plates with paired same standard strains served as negative controls. The cultures were initially incubated at 28 °C under light for 3 to 4 days and then transferred to 19 °C with light before mycelial contact occurred. After 25 days of cross-culture, if the test isolate and standard strain form perithecia at the intersection, the test isolate is considered as fertile, otherwise, as sterile. 20 perithecia were randomly selected from each isolate’s culture dish and placed on water agar (WA) medium. To ascertain the maturity of perithecia, we determined whether ascospores were released after crushing the perithecia with a needle under a stereomicroscope (Leica S8APO, Wetzlar, Germany), and the number of mature perithecia was recorded. Five mature perithecia were randomly selected from dishes containing mature perithecia. The collected perithecia were placed on a sterilized glass slide and covered with a sterilized coverslip, followed by gentle pressing. After removing the coverslip, 250 μL of sterile water was added to the slide to release ascospores from the crushed perithecia. The ascospore suspension was then collected and spread evenly on the surface of WA, and the ascospore germination rate was counted after 36 h. During scoring perithecia and ascospores, assessing the maturity, and counting ascospore germination, the samples were performed blindly to reduce observer bias.

### 2.4. Statistics

The correlation analysis (Pearson) and difference analysis (Duncan) were performed by using SPSS 24, BoxPlot, and OmicStudio.

## 3. Results

### 3.1. Geographical Distribution of Mating Types in M. oryzae

**Figure 1:**
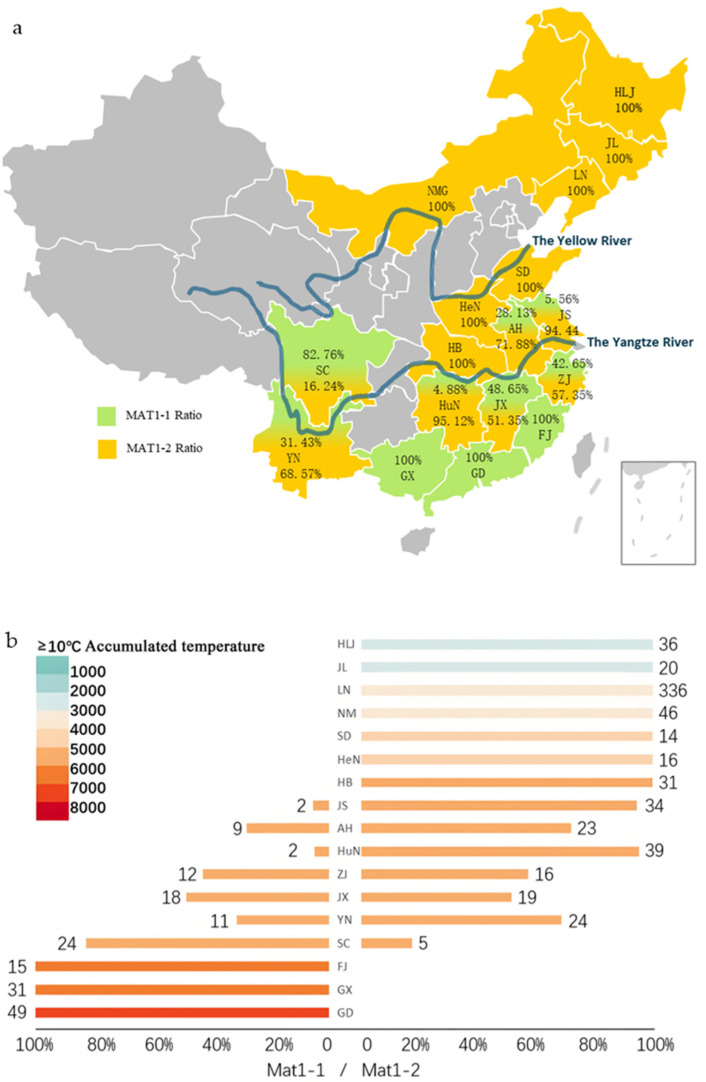
(**a**) The distribution of rice blast pathogen (*Magnaporthe oryzae)* collection sites and mating types in 17 provinces of China, where the proportion of green and yellow in a certain province represents the proportion of *MAT1-1* and *MAT1-2*, respectively. (**b**) The effective accumulated temperature and mating-type distribution of 17 provinces in China where the rice blast pathogen was collected were tested. Different colors represent different effective accumulated temperatures (≥10 °C), with the horizontal axis representing the proportion of two mating types (*MAT1-1* and *MAT1-2*). The number represents the number of isolates of the two mating types, and the vertical axis representing different provinces, including Heilongjiang (HLJ), Jilin (JL), Liaoning (LN), Neimenggu (NMG), Shandong (SD), Jiangsu (JS), Anhui (AH), Zhejiang (ZJ), Jiangxi (JX), Fujian (FJ), Henan (HeN), Hebei (HB), Hunan (HuN), Sichuan (SC), Yunnan (YN), Guangdong (GD) and Guangxi (GX). The gray areas indicate regions not covered in this study.

**Figure A1:**
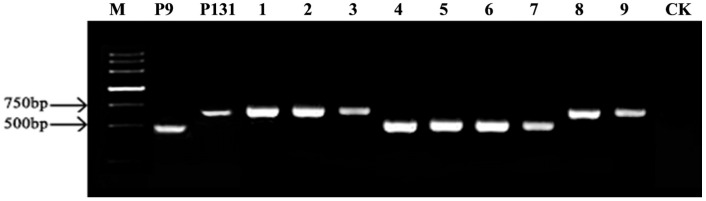
Electrophoresis mating-type detection of certain tested *Magnaporthe oryzae* isolates collected across China. P9 was utilized as the *MAT1-1* standard strain, and P131 was utilized as the *MAT1-2* standard strain. 1–9 lines were the random testing isolates. CK represents the negative control, and other lines indicate randomly selected tested isolates.

Mating type-specific primers were employed to obtain the geographical distribution of mating types among the tested isolate. The results showed that the specific primers *MAT1-1* and *MAT1-2* successfully amplified the target mating-type gene marker fragments in the standard strains P9 and P131, respectively (Figure A1), confirming the effectiveness of these primers for identifying *M. oryzae* mating-types. Molecular detection results from the 832 tested isolates revealed 173 isolates (20.79%) of *MAT1-1* and 659 isolates (79.21%) of *MAT1-2* (Figure 1; Table A1), indicating a significant predominance of the *MAT1-2* type within Chinese *M. oryzae* populations.

The examination of regional distribution characteristics revealed that NEC and NC exclusively harbored the *MAT1-2* type, while the SC population was exclusively composed of the *MAT1-1* type. CC was dominated by *MAT1-2* (97.73%), exhibiting a single mating-type pattern. In contrast, the population of EC consisted of 65.43% *MAT1-2*, and SWC exhibited mating type diversity, with *MAT1-1* and *MAT1-2* accounting for 54.69% and 45.31% of the population, respectively (Table A1). Overall, the geographical heterogeneity of *M. oryzae* mating types across Chinese rice-growing regions was notably high. At the provincial level, mating-type distributions in most provinces were predominantly single, with the populations of 10 provinces consisting of a single mating-type (Figure 1; Table A1). Among these provinces, seven provinces, including HLJ, JL, and LN, exclusively harbored the *MAT1-2* mating-type, while three provinces, including FJ, GD, and GX, exclusively harbored *MAT1-1*. Additionally, the dominant mating type exceeded 90% in two provinces; JS consisted of 94.44% *MAT1-2*, while the HuN population consisted of 95.12% *MAT1-2*. These 13 provinces all showed significant mating-type bias. In contrast, ZJ (*MAT1-1*: 42.86%, *MAT1-2*: 57.14%) and JX (*MAT1-1*: 48.65%, *MAT1-2*: 51.35%) exhibited nearly balanced distributions. From a geographical perspective, northern rice-producing regions and those along the Yellow River were characterized by a single mating-type, predominantly *MAT1-2*. The Yangtze River basin populations mainly consisted of mating types, while the southern coastal regions were exclusively occupied by single mating types.

**Figure 2:**
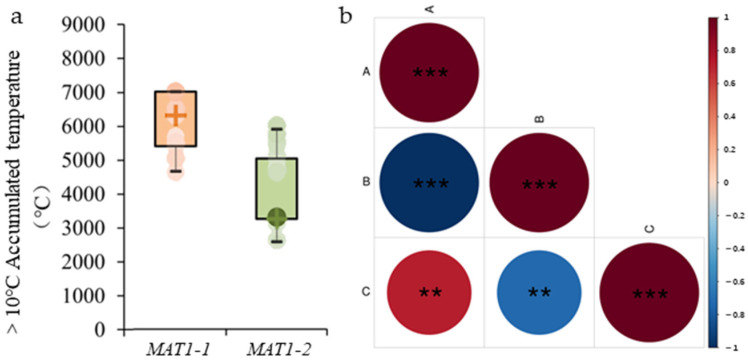
Correlation analysis between mating-type and effective accumulated temperature (≥10 °C) of *Magnaporthe oryzae*. (**a**) Histogram representing the distribution of two mating-types of *M. oryzae* within the effective accumulated temperature range of 0–9000 °C, where “+” is the median. The orange part is *MAT1-1*, and the green part is *MAT1-2* (**b**) Correlation between two mating-types of *M. oryzae* and effective accumulated temperature (≥10 °C). A represents the proportion of *MAT1-1* mating-type, B represents the proportion of *MAT1-2* mating-type, and C represents the effective accumulated temperature (≥10 °C). The horizontal and vertical intersections indicate the correlation between the two parameters, with the degree of correlation between color depth and circular area meeting the standard. Different shades and area of red indicate a positive correlation, while different shades and area of blue indicate a negative correlation. ** denotes significance at *p* < 0.01 and *** denotes significance at *p* < 0.001.

Analysis based on effective accumulated temperatures (≥10 °C) (data source: map of ≥10 °C accumulated temperatures in China from the China Geographic Atlas, 1 November 2009, online map is https://www.osgeo.cn/map/m0118/ (8 October 2025)) indicated that the *MAT1-1* mating type was primarily concentrated in the 4500–7000 °C accumulated temperature zone, whereas *MAT1-2* was mainly found in the 2500–5000 °C zone (Figure 2a). Correlation analysis revealed a significant relationship between *M. oryzae* mating-type and effective accumulated temperature (≥10 °C) (*p* < 0.01) (Figure 2b). These findings suggest that the effective accumulated temperature (≥10 °C) significantly influences the mating-type distribution of *M. oryzae* in China.

### 3.2. Variation in Fertility of M. oryzae

**Figure 3:**
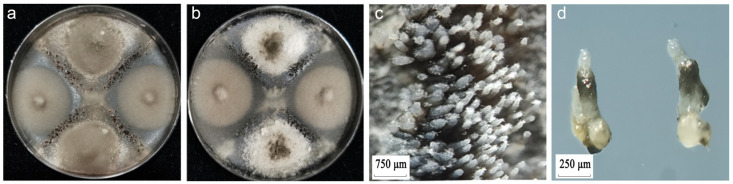
Morphological formation of *Magnaporthe oryzae*. (**a**) Perithecia formed via the hybridization of standard strains P9 (*MAT1-1*) and P131 (*MAT1-2*). (**b**) Perithecia formed via the hybridization of hermaphroditic strains (P9 × DG10). (**c**) Top view of perithecia produced through sexual hybridization between opposite mating-type strains. (**d**) Longitudinal diagram of mature ascocarp morphology.

The fertility of *M. oryzae* was assessed using the cross-culture method. After approximately 25 days of culture, the standard strains P9 (*MAT1-1*) and P131 (*MAT1-2*) produced perithecia (Figure 3a), and the same standard strain could not produce perithecia. The growth rates of the mycelium of the two standard strains are basically the same. Some tested isolates generated perithecia under these conditions (Figure 3b). Visible black dot-like bands formed at the mycelial intersection. Stereomicroscope observations further revealed numerous perithecia with translucent hairy tops at the mycelial intersection. The perithecia were semi-embedded in the medium surface, and mature perithecia exhibited long beaks protruding from the surface of the medium (Figure 3c,d). These observations confirm the effectiveness of this method for assessing the fertility of *M. oryzae*.

**Figure 4:**
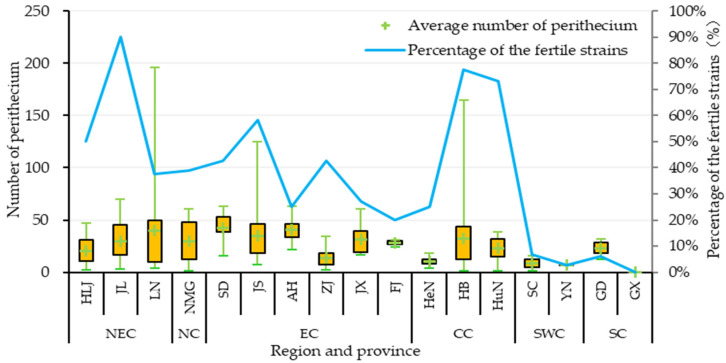
Variations in the fertility of *Magnaporthe oryzae* across different regions of China. The horizontal axis represents different provinces; the left vertical axis represents the number of perithecia produced by fertile isolates, the “+” in the quartile chart indicates the median; and the right vertical axis shows the percentage of fertile isolates among the tested isolates in the province.

The fertility test results for the tested isolates (Figure 4, Table A2) revealed that only 304 out of 832 isolates could produce perithecia with the standard strains, representing a fertility rate of 36.54%. Among the fertile isolates, 21 isolates successfully hybridized with P131, accounting for 12.14% (21/173) of the total *MAT1-1* isolates, while 283 isolates successfully hybridized with P9, accounting for 42.94% (283/659) of the total *MAT1-2* isolates. This indicates that field *MAT1-2* isolates exhibit higher fertility than *MAT1-1* isolates.

The proportion of fertile isolates varied considerably across different regions of China, ranging from 3.75% to 65.91% (Table A2). The fertile proportions of three regions exceeded the average, with that of CC (65.91%) being the highest, followed by that of NEC (41.33%) and NC (39.13%). Three regions fell below the average, with EC (37.04%) being slightly below, followed by SWC (4.69%), and SC (3.75%) harboring the lowest proportion of fertile isolates. The difference in fertile isolates proportion among the three provinces in the CC region was the largest (52.42%; from HeN: 25.00% to HB: 77.42%), while the difference between the two provinces in the SWC region was the smallest (4.04%; from YN: 2.86% to SC: 6.90%). Significant variation in fertile isolates proportion was also observed among different provinces, ranging from 0.00% to 90.00%. The fertile proportions in nine provinces exceeded the average level (36.54%), with JL (90.00%) being the highest, followed by HB (77.42%) and HuN (73.17%). The fertile proportions of a further nine provinces fell below the average; YN had only 2.86% fertile isolates, while all 31 isolates isolated from GX were sterile. These results indicate that while fertility varies across different regions in China, the overall fertility level of the *M. oryzae* population is relatively low.

**Figure A2:**
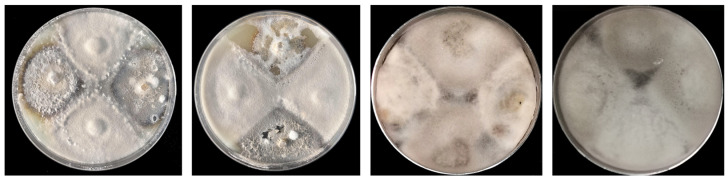
Schematic diagram of confrontation cultivation of selected *Magnaporthe oryzae* isolates collected from JX and ZJ provinces. *MAT1-1* and *MAT1-2* isolates collected from within each province were hybridized with each other, resulting in a total of 516 combinations, among which no combinations produced sexual states.

To examine whether the basic conditions for sexual generation exist within a single region, 37 and 28 tested isolates from JX and ZJ provinces, respectively, which exhibit mating-type ratios of close to 1:1 and thus provide optimal natural conditions for the two mating types to encounter each other, were selected for intra-regional hybridization tests. These isolates were subjected to cross-culture in 516 combinations. The results showed that none of the paired cultures produced any perithecia; however, this includes 10 isolates of *MAT1-1* and 12 isolates of *MAT1-2 M. oryzae* that can be produced perithecia with standard isolates (Figure A2).

Further analysis of the capacity for sexual reproduction in *M. oryzae* revealed significant differences in perithecium production capacity among different isolates (Figure 4; Table A2). Among the 304 fertile isolates, the number of perithecia formed ranged from 3 to 196, with an average of 26.0. The maximum value was observed in isolates collected from the LN province in NEC. The number of isolates producing 1–50 perithecia was the highest (245 isolates, 80.59% of fertile isolates), followed by 29 isolates producing 51–100 perithecia (9.54% of fertile isolates). Isolates producing more than 100 perithecia accounted for 9.87%. The average number of perithecia per isolate varied across different regions, with EC (32.0) having the highest number and SWC (8.3) having the lowest number. Differences were also observed among provinces, with SD (42.8) having the highest average, followed by ZJ (40.9), and YN (7.0) having the lowest average. Overall, JL had the highest proportion of fertile isolates (90.00%), with an average perithecium count of 29.8 per fertile isolate. SD had the highest average perithecium count per fertile isolate (42.8), with a fertility rate of 42.86%.

Further analysis of perithecium maturity revealed small differences in the mature perithecium and ascospore germination rates across regions. The mature perithecium rate was the lowest in ZJ (73.33%) and the highest in JX (93.33%). The ascospore germination rate was the lowest in SD (8.33%) and the highest in YN (21.15%) (Table A2).

### 3.3. Correlation Analysis Between Mating-Type and Fertility in M. oryzae

**Figure 5:**
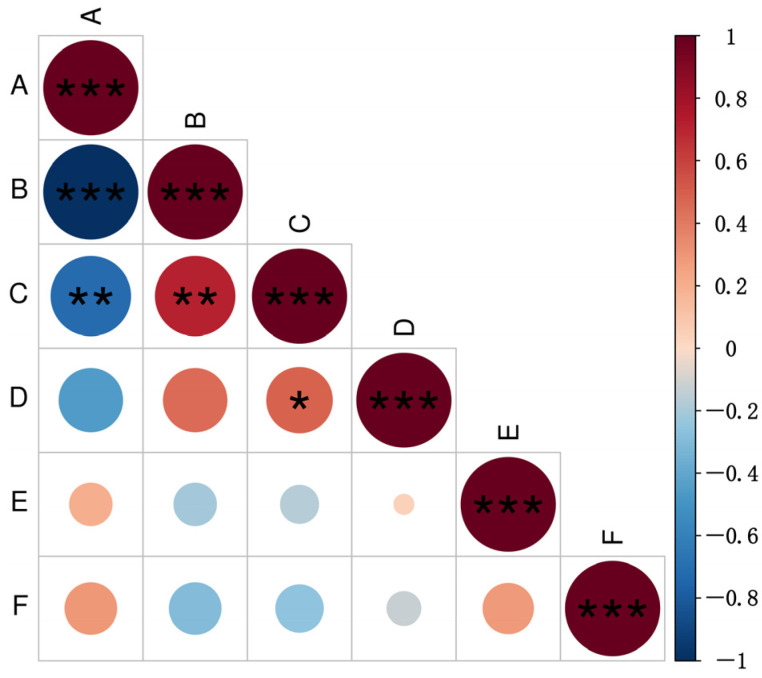
Analysis of correlations between mating type and fertility in *Magnaporthe oryzae*. A: proportion of the *MAT1-1* mating-type; B: proportion of the *MAT1-2* mating-type; C: proportion of fertile isolates; D: number of perithecia produced; E: mature perithecium rate; F: ascospore germination rate. The horizontal and vertical intersections indicate the correlation between the two parameters, with the degree of correlation between color depth and circular area meeting the standard. Different shades of red indicate a positive correlation, while different shades of blue indicate a negative correlation. * denotes significance at *p* < 0.05, ** denotes significance at *p* < 0.01, and *** denotes significance at *p* < 0.001.

Correlation analysis revealed a highly significant negative correlation between fertility and the *MAT1-1* mating type (*p* < 0.01), whereas a highly significant positive correlation was detected between fertility and the *MAT1-2* mating type (*p* < 0.01) (Figure 5). Furthermore, the number of fertile isolates per region exhibited a significant positive correlation with the average number of perithecia formed (*p* < 0.05). These results suggest a certain association between fertility and mating-type in *M. oryzae*, consistent with the above results that *MAT1-2* isolates exhibit higher fertility than *MAT1-1* isolates. Furthermore, there is no significant correlation between mating type, mature perithecium rate and ascospore germination rate.

## 4. Discussion

To date, the perfect stage of *M. oryzae* has not been observed on various rice organs (including leaves, stems, and panicles) under natural conditions [21,22,23]. Nevertheless, sexual recombination is widely considered as a significant potential mechanism for its genetic variation [24,25]. This study systematically analyzed the distribution characteristics of mating types and differences in sexual reproductive ability in *M. oryzae* populations through laboratory experiments. The results revealed that populations in geographically isolated regions were generally dominated by a single mating type, resulting in a lack of the complementary mating types necessary for sexual reproduction within local populations. This finding is in accordance with previous research results [9,26,27,28]. Notably, although isolates collected from various rice-growing regions could successfully hybridize with standard strains P9 or P131 to produce a perfect stage in the laboratory, no perithecia was generated through interactions among isolates collected from the same geographic population [29,30,31,32,33]. These results suggest that, under natural ecological conditions, *M. oryzae* struggles to meet the spatiotemporal matching requirements for sexual reproduction. Consequently, population maintenance and dispersal in *M. oryzae* primarily depend on asexual reproduction.

Among the tested isolates, 336 isolates collected from LN encompassing diverse geographical forms including coastal, riverside, mountainous, and plain areas, with evident environmental differences and varietal growth periods ranging from 130 to 160 days—all exhibited the *MAT1-2* mating type. This suggests that topographic factors and varietal differences are not the primary drivers of mating-type deviation in *M. oryzae*. Correlation analysis between mating-types from 832 isolates and geographical environmental parameters revealed that the distribution of mating types in *M. oryzae* across China exhibited distinct regional characteristics, with spatial differentiation closely associated with the effective accumulated temperature (≥10 °C). China’s vast north–south span covers effective accumulated temperature zones that range from 2500 °C in the north to 7000 °C in the south. The results of the present study demonstrate that the proportion of the *MAT1-1* mating type in *M. oryzae* increases correspondingly, with southern coastal regions exclusively harboring *MAT1-1* isolates. The mating type is affected by accumulated temperature, which may be due to differences in rice varieties planted in the north and the south, as well as the strong cold-resistance of *MAT1-2* rice blast fungus in winter to help the fungi overwinter.

Mating-type imbalance is also commonly observed in other fungal species, such as *Botrytis cinerea* and *Bipolaris maydis* [34,35]. One possible explanation is a potential linkage between a specific mating type and mutated fertility-inhibiting factors carrying lethal effects [15]. This linkage may cause the segregation ratio of mating types in perithecia to deviate from 1:1, gradually leading to a population-wide imbalance in the mating-type ratio. However, mating type does not affect the occurrence and prevalence of the rice blast fungus. For example, 336 *M. oryzae* isolates collected from Liaoning Province were all *MAT1-2* type, but the degree of occurrence of rice blast on susceptible cultivar ‘LTH’ in different collection locations was significantly different, and it was more influenced by climatic factors. Given that the wild-type *M. oryzae* mainly reproduces asexually and has a relatively low degree of variation, changes on fungal virulence may be attributed by environmental pressures, such as long-term and large-scale cultivation of a few rice varieties.

Fertility in *M. oryzae* is controlled by multiple genetic loci [36]. The present study identified a correlation between fertility and mating types, suggesting a potential genetic linkage between the mating-type gene and fertility-related genetic loci though it remain unclear. Additionally, even under artificially favorable conditions, including encounters between different mating types and suitable temperatures, sexual reproduction was rarely achieved between wild and standard strains, while no sexual reproduction was observed between two wild isolates. However, in our study we did not determine whether the sexual sterile was caused by hyphal incompatibility or post-mating developmental failure. These results imply that in natural environments lacking such conditions, the likelihood of *M. oryzae* undergoing sexual reproduction is substantially reduced.

## 5. Conclusions

In summary, the results of this study demonstrate that the *M. oryzae* population in China’s major rice-growing regions is predominantly characterized by the *MAT1-2* mating type (79.21%). However, significant mating-type deviation was observed in most provinces. A notable correlation was identified between mating type and the effective accumulated temperature (≥10 °C), with *MAT1-1* being mainly concentrated in the 4500–7000 °C zone, while *MAT1-2* is primarily found in the 2500–5000 °C zone. Under artificial conditions, when hybridized with standard strains, the proportion of fertile wild isolates was relatively low (36.54% = 304/832), and *MAT1-2* isolates (42.94%) exhibited higher fertility compared to *MAT1-1* isolates (12.14%). However, when isolates of opposite mating-types isolated from the same region were cross-culture under controlled laboratory conditions, none of the combinations produced perithecia. Therefore, sexual reproduction is rare or absent in *M. oryzae* populations under natural conditions in China’s main rice-producing areas, with the pathogen populations primarily consisting of asexual lineages. To the best of our knowledge, this work represents the most comprehensive report to date on the mating-type and fertility assessment of *M. oryzae* in China. However, the possibility of rare occurrences of the minority mating-type in provinces currently dominated by a single type cannot be ruled out. Given the dynamic nature of *M. oryzae* populations and inter-regional exchange, both mating types should continue to be monitored.

## Data Availability

The data is not publicly available due to the status of collaborative conformity.

## Appendix A

**Table A1:** The mating types of *Magnaporthe oryzae* in China.

Region	Province	Accumulated Temperature	No. of Isolates	*MAT1-1*		*MAT1-2*	
		≥10 °C		Quantity	Proportion	Quantity	Proportion
NEC	HLJ	2500	36	0	0.00%	36	100.00%
	JL	2500	20	0	0.00%	20	100.00%
	LN	3270	336	0	0.00%	336	100.00%
NC	NM	3100	46	0	0.00%	46	100.00%
EC	SD	4570	14	0	0.00%	14	100.00%
	JS	5050	36	2	5.56%	34	94.44%
	AH	4950	32	9	28.12%	23	71.88%
	ZJ	5500	28	12	42.86%	16	57.14%
	JX	5500	37	18	48.65%	19	51.35%
	FJ	6300	15	15	100.00%	0	0.00%
CC	HeN	4700	16	0	0.00%	16	100.00%
	HB	5400	31	0	0.00%	31	100.00%
	HuN	5700	41	2	4.88%	39	95.12%
SWC	SC	5050	29	24	82.76%	5	17.24%
	YN	5400	35	11	31.43%	24	68.57%
SC	GD	7000	49	49	100.00%	0	0.00%
	GX	6500	31	31	100.00%	0	0.00%
Total		-	832	173	20.79%	659	79.21%

**Table A2:** The Fertility variation in Magnaporthe *oryzae* in China.

Region	Province	No. of Isolates	Fertile Isolates			Perithecia			Mature Perithecium Rate	Spore Germination Rate
			Quantity	Proportion		1–50	51–100	>100		
NEC	HLJ	36	18	50.00%	41.33%	18	0	0	86.67%(13/15)	14.42%(15/104)
	JL	20	18	90.00%		15	0	3	80.00%(12/15)	15.63%(15/96)
	LN	336	126	37.50%		96	16	14	86.67%(13/15)	18.10%(19/105)
NC	NM	46	18	39.13%	39.13%	14	3	1	80.00%(12/15)	16.33%(16/98)
EC	SD	14	6	42.86%	37.04%	2	0	4	80.00%(12/15)	8.33%(8/96)
	JS	36	21	58.33%		16	3	2	86.67%(13/15)	13.73%(14/102)
	AH	32	8	25.00%		6	1	1	80.00%(12/15)	15.46%(15/97)
	ZJ	28	12	42.86%		9	0	3	73.33%(11/15)	14.74%(14/95)
	JX	37	10	27.03%		8	2	0	93.33%(14/15)	16.96%(19/112)
	FJ	15	3	20.00%		3	0	0	80.00%(12/15)	13.13%(13/99)
CC	HeN	16	4	25.00%	65.91%	3	0	1	80.00%(12/15)	13.86%(14/101)
	HB	31	24	77.42%		20	4	0	86.67%(13/15)	20.19%(21/104)
	HuN	41	30	73.17%		29	0	1	80.00%(12/15)	14.29%(14/98)
SWC	SC	29	2	6.90%	4.69%	2	0	0	86.67%(13/15)	18.63%(19/102)
	YN	35	1	2.86%		1	0	0	86.67%(13/15)	21.15%(22/104)
SC	GD	49	3	6.12%	3.75%	3	0	0	80.00%(12/15)	18.75%(18/96)
	GX	31	0	0.00%		-	-	-	-	-
Total		832	304	36.54%		245	29	30	-	-

**Table A3:** PCR primers for mating-type detection of *Magnaporthe oryzae* [20].

Primer	Sequences (5′ to 3′)
MAT1-1F	AGCCTACTACGCTGGCATCT
MAT1-1R	GGTGACAGAGCTGTCTTCCA
MAT1-2F	GCATCAACCAGGTCTCAGTC
MAT1-2R	ATCCTCAGGTTCATCGACAG
